# Production of the Antimicrobial Roseoflavin With Genetically Engineered *Corynebacterium glutamicum*

**DOI:** 10.1111/1751-7915.70246

**Published:** 2025-10-22

**Authors:** Luciana Fernandes Brito, Ane Bræin Aas, Rosa Jodalen Rudberg, Trygve Brautaset, Fernando Pérez‐García

**Affiliations:** ^1^ Department of Biotechnology and Food Science Faculty of Natural Sciences, NTNU Trondheim Norway

**Keywords:** antimicrobial, *Corynebacterium glutamicum*, metabolic engineering, *ribM*, roseoflavin

## Abstract

The global rise of drug‐resistant bacterial infections underscores an urgent demand for novel antimicrobial agents. Roseoflavin, a naturally occurring riboflavin analog, has emerged as a promising candidate for drug development. In this study, the industrial workhorse *Corynebacterium glutamicum* was metabolically engineered towards roseoflavin production. Overexpression of the roseoflavin biosynthetic genes *rosABC* and the flavin transport gene *ribM* from the native roseoflavin producer *Streptomyces davaonensis* was evaluated in *C. glutamicum* . To further link roseoflavin biosynthesis to its riboflavin precursor, the riboflavin kinase gene *ribF* from both *S. davaonensis* and *C. glutamicum* was evaluated, revealing that its overexpression is essential for enhancing roseoflavin production. The final engineered strain CgRose6 achieved a roseoflavin titer of 17.4 ± 1.5 mg/L and a volumetric productivity of 0.36 ± 0.03 mg/L·h when cultivated in glucose minimal medium supplemented with thiamine, a relevant coenzyme for roseoflavin biosynthesis. These production values are the highest reported by a non‐native RoF producer to date and demonstrate the feasibility of using *C. glutamicum* as a platform for sustainable roseoflavin production, opening avenues for scalable biosynthesis of this valuable antimicrobial compound.

## Graphical abstract

*Corynebacterium glutamicum* was genetically engineered for the production of the riboflavin analog roseoflavin, while minimising its antimicrobial effects. To this end, the genetic machinery from the natural roseoflavin producer *Streptomyces davaonensis* was evaluated in a riboflavin‐producing *C. glutamicum* strain. Thiamine supplementation in the medium further improved roseoflavin biosynthesis.

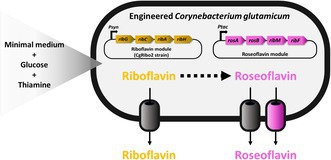

## 1 Introduction

Antibiotic resistance has emerged as a major public health concern of the 21st century, presenting a growing threat and attracting increasing attention. As a result, there is an urgent need for new antimicrobial agents to combat multidrug‐resistant bacterial pathogens (Charoenwongpaiboon et al. 2023; Lou et al. 2025). Roseoflavin (RoF) is a naturally occurring analogue of riboflavin (RF), which exhibits notable antimicrobial activity against several Gram‐positive bacteria, including *Staphylococcus aureus* and *Bacillus subtilis* . However, its effectiveness against Gram‐negative bacteria is limited, primarily due to restricted RoF uptake in these organisms (Mansjö and Johansson 2011; Pedrolli et al. 2012). RoF is produced by the soil bacteria *Streptomyces davaonensis* (formerly *Streptomyces davawensis*) and *Streptomyces cinnabarinus*, both Gram‐positive, filamentous and resistant to RoF (Mora‐Lugo et al. 2019). RoF shares a close structural similarity with RF, differing primarily by the presence of an additional dimethylamine group. This resemblance allows RF transporters to import RoF into the cytosol of Gram‐positive bacteria (Gutiérrez‐Preciado et al. 2015). Inside bacteria, promiscuous flavokinase enzymes facilitate the conversion of RoF to roseoflavin mononucleotide (RoFMN), while FAD synthetase catalyses the formation of roseoflavin adenine dinucleotide (RoFAD) from RoF (Grill et al. 2008). The occurrence of those modified flavins within the bacterial environment disrupts the function of flavoproteins, as RoFMN and RoFAD take the place of flavin mononucleotide (FMN) and flavin adenine dinucleotide (FAD) in the binding sites of these proteins (Langer et al. 2013). The essential role of flavoproteins in cell function and growth is compromised, leading to the manifestation of the toxic properties of RoF. Additionally, RoFMN induces feedback inhibition on RF production by binding to the FMN riboswitch (Kißling et al. 2020). FMN riboswitches play a regulatory role in governing the expression of genes involved in both the biosynthesis and transport of RF, responding to the levels of FMN (Mora‐Lugo et al. 2019). Upon binding to the binding sites of these riboswitches and displacing FMN, RoFMN interrupts the production of RF (Mora‐Lugo et al. 2019). Considering the dependence of bacteria on flavoprotein function, coupled with the prevalence of FMN riboswitches in many bacterial species, RoF is acknowledged as a promising antimicrobial compound directed against medically relevant bacteria (Mora‐Lugo et al. 2019). For instance, RoF has been shown to prevent the growth of *S. aureus* , *Enterococcus faecalis* and *Listeria monocytogenes* (Charoenwongpaiboon et al. 2023). Additionally, RoF is utilised in the food industry for RoF‐resistant strain selection to produce RF‐enriched bread and pasta (Russo et al. 2014).

**FIGURE 1:**
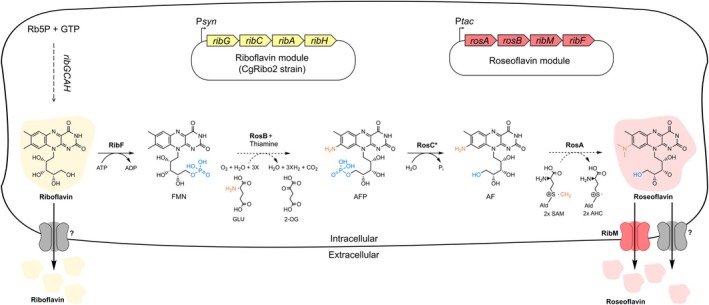
Metabolic pathway for conversion of the vitamin riboflavin (RF) to the antimicrobial roseoflavin (RoF) in engineered *C. glutamicum* . The RF genetic module (P*syn*‐*ribGCAH*) was previously developed to establish RF overproduction in *C. glutamicum* through the constitutive overexpression of its native RF biosynthetic genes, resulting in the strain CgRibo2 (Pérez‐García et al. 2022). The RoF genetic module (P*tac*‐*rosAB‐ribMF*) developed in this study enabled the inducible conversion of RF to RoF and the secretion of the latter as a defense mechanism against its own antimicrobial effects. The metabolites depicted in the figure are as follows: Ri5P, ribulose‐5‐phosphate; GTP, guanosine triphosphate; FMN, flavin mononucleotide; AFP, 8‐demethyl‐8‐amino‐riboflavin‐5′‐phosphate; AF, 8‐demethyl‐8‐amino‐riboflavin; ATP, adenosine triphosphate; ADP, adenosine diphosphate; Thi, thiamine; Glu, glutamate; Oxo, 2‐oxoglutarate; Pi, inorganic phosphate; SAM, *S*‐adenosylmethionine; SAH, *S*‐adenosylhomocysteine. The enzymes/proteins involved in the pathway are: RibF, bifunctional RF kinase/FMN adenylyltransferase; RosA, SAM‐dependent AF dimethyltransferase; RosB, AFP synthase; RosC, AFP phosphatase (* based on our results, this reaction is not essential under the conditions tested); RibM, flavin transport protein.

Currently, RoF is industrially produced via chemical synthesis, a costly process that involves hazardous compounds, posing significant health risks. Starting from N,N‐dimethyl‐o‐toluidine, this multi‐step procedure results in low molar yields of about 5% RoF (Mora‐Lugo et al. 2019; Otani et al. 1980). Therefore, there is a pressing need for more sustainable methods of RoF production. The biosynthesis of RoF in *S. davaonensis* and *S*. *cinnabarinus* starts with RF. The bifunctional enzyme RF kinase, also known as FMN adenylyltransferase (encoded by *ribF*), catalyses the phosphorylation of RF to produce riboflavin‐5′‐phosphate, using ATP as the phosphate donor (Schneider et al. 2020). Subsequently, 8‐demethyl‐8‐amino‐riboflavin‐5′‐phosphate synthase (encoded by *rosB*) converts this intermediate into 8‐demethyl‐8‐amino‐riboflavin‐5′‐phosphate (AFP) through a series of enzymatic reactions, with thiamine serving as a coenzyme. AFP is then dephosphorylated by AFP phosphatase (encoded by *rosC*) in the presence of water to form 8‐demethyl‐8‐amino‐riboflavin (AF) (Schneider et al. 2020). Finally, using *S*‐adenosylmethionine (SAM) as the methyl donor, the SAM‐dependent dimethyltransferase (encoded by *rosA*) catalyses the formation of RoF through two methylation steps (Schneider et al. 2020) (Figure 1). In *S. davaonensis*, the membrane protein encoded by *ribM* facilitates the import of RF and the export of RoF, serving as a defense mechanism against RoF's toxic effects (Hemberger et al. 2011). However, the natural RoF producers S. *davaonensis* and *S. cinnabarinus* are not suitable for industrial bioproduction, as they tend to adhere to surfaces and grow in aggregates, hampering cultivation in bioreactors (Mora‐Lugo et al. 2019). Consequently, bioprocess‐based production of RoF presents significant challenges, making the search for a more suitable microbial host an appealing and necessary alternative. Therefore, engineering robust and well‐characterised bacterial strains is essential to overcome these limitations and enable efficient, scalable RoF production.

Hence, in this study, we aimed to develop an alternative approach for RoF production through metabolic engineering of the industrial workhorse *Corynebacterium glutamicum* . Our previously engineered an RF‐overproducing *C. glutamicum* strain CgRibo2 (Pérez‐García et al. 2022) laid the foundation for this work. We assessed the toxic effects of RoF on the *C. glutamicum* wild‐type strain, an RF‐producing *C. glutamicum* strain and *C. glutamicum* expressing the *ribM* gene from *S. davaonensis*, which encodes a RF/RoF transporter. Additionally, we introduced and evaluated the RoF biosynthetic genes *rosABC* from *S. davaonensis*, both with and without co‐expression of *ribM*. Furthermore, the bifunctional RF kinase (*ribF*) genes from *C. glutamicum* and *S. davaonensis* were evaluated for their impact on RF and RoF production (Figure 1). Finally, the best‐performing engineered CgRose6 strain was tested in minimal medium supplemented with relevant cofactors. By systematically constructing a synthetic RoF biosynthesis pathway in *C. glutamicum* , this study reports the highest RoF titer achieved by a non‐native RoF producer under small‐scale flask conditions to date.

## 2 Materials and Methods

### 2.1 Bacterial Strains, Plasmids and Growth Conditions

Table 1 provides an overview of all bacterial strains and plasmids utilised in this study. *Escherichia coli* DH5α was grown at 37°C using lysogeny broth (LB; consisting of 10 g/L tryptone, 5 g/L yeast extract and 5 g/L NaCl) or on solid LB agar plates. For *C. glutamicum* precultures, 2YT medium (containing 16 g/L tryptone, 10 g/L yeast extract and 5 g/L NaCl) or 2YT agar plates were used, with incubation at 30°C. Main cultures of *C. glutamicum* were performed in 500 mL Erlenmeyer shake flasks with a working volume of 10% (50 mL) of CGXII minimal medium as described by Eggeling et al. (Eggeling et al. 2005), starting at an initial optical density at 600 nm (OD_600_) of approximately 1, and supplemented with 10 g/L glucose as the sole carbon source. The OD_600_ values were measured using an Ultrospec 7500 (Biochrom Ltd.). When required, the culture medium was supplemented with kanamycin at a final concentration of 25 μg/mL and/or tetracycline at 5 μg/mL. For gene expression from plasmid pVWEx1 (Peters‐Wendisch et al. 2001), 1 mM isopropyl‐β‐D‐1‐thiogalactopyranoside (IPTG) was added to the medium to induce transcription. To quantify RF and RoF, culture samples were collected, centrifuged at 16,000 × g and 4°C to separate the cells, and the resulting supernatants were stored at −20°C until analysis. Glucose, antibiotics and biotin were sterilised by filtration, whereas all other media components were sterilised via autoclaving. Unless stated otherwise, all consumables and chemicals used in this study were obtained from Merck/Sigma.

**TABLE 1:** List of strains and plasmids used in this work.

Strain or plasmid name	Description	Source
Strains		
*Escherichia coli* DH5α	*∆lac*U169 (φ80*lacZ* ∆M15), *sup*E44, *hsd*R17, *rec*A1, *end*A1, *gyr*A96, *thi*‐1, *rel*A1	(Hanahan 1983)
*Corynebacterium glutamicum*	Wild‐type strain ATCC 13032	(Abe et al. 1967)
CgRibo2	*C. glutamicum* carrying the plasmid pECXT‐*Psyn‐ribGCAH*. RF overproducer.	(Pérez‐García et al. 2022)
*C. glutamicum* (pVWEx1‐*ribM*)	*C. glutamicum* carrying the plasmid pVWEx1‐*ribM*	This work
*C. glutamicum* (pVWEx1‐*ribF* ^*Cg*^)	*C. glutamicum* carrying the plasmid pVWEx1‐*ribF* ^*Cg*^	This work
*C. glutamicum* (pVWEx1‐*ribF* ^*Sda*^)	*C. glutamicum* carrying the plasmid pVWEx1‐*ribF* ^*Sda*^	This work
CgRose1	*CgRibo2* carrying the pVWEx1‐*rosAB*	This work
CgRose2	*CgRibo2* carrying the pVWEx1‐*rosAB*‐*ribM*	This work
CgRose3	*CgRibo2* carrying the pVWEx1‐*rosABC*	This work
CgRose4	*CgRibo2* carrying the pVWEx1‐*rosABC*‐*ribM*	This work
CgRose5	*CgRibo2* carrying the pVWEx1‐*rosAB*‐*ribM*‐*ribF* ^*Cg*^	This work
CgRose6	*CgRibo2* carrying the pVWEx1‐*rosAB*‐*ribM*‐*ribF* ^*Sda*^	This work
Plasmids		
pVWEx1	Kan^R^, *C. glutamicum* / *E. coli* shuttle plasmid (Ptac, *lacI*, pBL1 oriV_Cg_)	(Peters‐Wendisch et al. 2001)
pVWEx1‐*ribM*	Kan^R^, pVWEx1 overexpressing the gene *ribM* from *S. davaonensis*	This work
pVWEx1‐*rosAB*	Kan^R^, pVWEx1 overexpressing the genes *rosA* and *rosB* from *S. davaonensis*	This work
pVWEx1‐*rosAB‐ribM*	Kan^R^, pVWEx1 overexpressing the genes *rosA, rosB* and *ribM* from *S. davaonensis*	This work
pVWEx1‐*rosABC*	Kan^R^, pVWEx1 overexpressing the genes *rosA, rosB* and *rosC* from *S. davaonensis*	This work
pVWEx1‐*rosABC‐ribM*	Kan^R^, pVWEx1 overexpressing the genes *rosA, rosB, rosC* and *ribM* from *S. davaonensis*	This work
pVWEx1‐*rosAB‐ribM*‐*ribF* ^*Cg*^	Kan^R^, pVWEx1 overexpressing the genes *rosA, rosB and ribM* from *S. davaonensis* as well as the gene *ribF* from *C. glutamicum*	This work
pVWEx1‐*rosAB‐ribM*‐*ribF* ^*Sda*^	Kan^R^, pVWEx1 overexpressing the genes *rosA, rosB, ribM* and *ribF* from *S. davaonensis*	This work
pVWEx1‐*ribF* ^*Cg*^	Kan^R^, pVWEx1 overexpressing the gene *ribF* from *C. glutamicum*	This work
pVWEx1‐*ribF* ^*Sda*^	Kan^R^, pVWEx1 overexpressing the kinase *ribF* from *S. davaonensis*	This work

### 2.2 Molecular Genetic Techniques and Strain Construction

Molecular cloning procedures were carried out using established protocols (Green and Sambrook 2012). Polymerase chain reaction (PCR) for cloning purposes was routinely performed using the CloneAmp HiFi PCR Premix (Takara Bio Inc.), while colony PCR was performed using the GoTaq DNA Polymerase (Promega). *E. coli* DH5α served as the host strain for plasmid construction. Transformation of *E. coli* DH5α was achieved via the standard heat shock method (Green and Sambrook 2012), whereas *C. glutamicum* strains were transformed by electroporation using a single electrical pulse (2.5 kV, 200 Ω, 25 μF) following previously reported protocols (Eggeling et al. 2005). The genes *rosA* (BN159_8032), *rosB* (BN159_7989), *rosC* (BN159_8033), *ribM* (BN159_7145) and *ribF* ^*Sda*^ (BN159_2715) were amplified from genomic DNA of *S. davaonensis* DSM 101723; the gene *ribF* ^*Cg*^ (Cgl1978) was amplified from genomic DNA from *C. glutamicum* ATCC13032. Details of all primers, including their names, sequences and functions, are provided in Table S1. To construct the plasmids used in this study, pVWEx1 was digested with the restriction enzyme BamHI (New England Biolabs, NEB) and assembled with the respective PCR‐amplified fragments using Gibson Assembly (Gibson et al. 2009).

### 2.3 Antimicrobial Tests

To test the antimicrobial effect of RoF on *C. glutamicum* , microtiter plates (Nunc MicroWell 96‐Well Optical‐Bottom Plates, Thermo Scientific) were used to cultivate 200 μL cultures of the *C. glutamicum* strains in 0.5% glucose minimal medium containing 1 mM IPTG and supplemented with gradually increasing concentrations of RoF, ranging from 0 mg/L to 500 mg/L. Overnight 2YT precultures were used to inoculate the minimal medium to an initial OD_600_ of approximately 0.1. To provide maximal sensitivity to growth inhibition while avoiding stationary‐phase artifacts, the plates were incubated for 8 h at 30°C on an incubator shaker at 1000 rpm (Innova 42, Eppendorf). After incubation, optical density at 600 nm (OD600) was measured using a plate reader (Infinite 200 Pro, Tecan).

### 2.4 Kinase Activity Assay

To obtain crude extracts for enzymatic assays, *C. glutamicum* pellets were harvested during the mid‐exponential growth phase (OD₆₀₀ = 2.5–3.5) after cultivation in CGXII minimal medium. The pellets were resuspended in 1 mL of enzyme assay buffer and mixed with 200 μL of 0.1 mm diameter glass beads. Crude extracts containing the enzymes of interest were prepared using a mixer mill (MM400, Retsch). Four 2‐min grinding cycles were performed at a frequency of 30 1/s, with 2‐min incubation steps on ice between each cycle. Cell debris was removed by centrifugation for 1 h at 16,000 rpm and 30°C. To evaluate the RF and RoF kinase activities of the RibF enzymes analysed in this study, the Kinase Assay Kit (MAK441‐1KT, Sigma‐Aldrich) was employed. This assay relies on fluorescent detection of ADP produced during the enzymatic reaction. Each reaction well contained 40 μL of the kit‐supplied buffer, 4 μL of 4 mM ATP, 16 μL of either 4 mM RF or RoF, 80 μL of reagent A, 40 μL of reagent B and 20 μL of enzyme‐containing crude extracts. Provided ADP standards were prepared in a range from 0 to 10 μM, following the manufacturer's instructions. After a 10‐min incubation, fluorescence was recorded with excitation at 530 nm and emission at 590 nm on a plate reader (Infinite 200 Pro, Tecan). Specific activity was calculated by normalising the volumetric activities to the protein concentrations (U/mg), where volumetric enzyme activities were expressed as μM of ADP produced per minute per mL (U/mL). Protein levels were quantified using the Bradford method (Bio‐Rad), with bovine serum albumin standards ranging from 1 to 0.063 mg/mL (Bradford 1976).

### 2.5 Analytical Procedures

Extracellular RF was quantified using a Waters Alliance e2695 HPLC system. Culture supernatants were first diluted 1:20 and stored at −20°C prior to analysis. Chromatographic separation was carried out on a Symmetry C18 column (75 × 4.6 mm, 3.5 μm; Waters), maintained at 25°C. Detection was achieved via a fluorescence detector (2475 FLR Detector, Waters), with excitation and emission wavelengths set to 370 and 520 nm, respectively. The mobile phase consisted of 5 mM ammonium acetate (pH 6.0) and methanol in a 3:1 ratio, delivered at a flow rate of 0.8 mL/min. Sample preparation was performed by mixing 500 μL of diluted supernatant with 1 mL of trichloroacetic acid to remove proteins, followed by incubation in the dark at 25°C for 20 min. The mixtures were then centrifuged at 8000 rpm for 20 min at 4°C. From the resulting supernatants, 1 mL was combined with 150 μL of 2 M potassium phosphate buffer (K₃PO₄) for pH adjustment before transferring to HPLC vials for analysis. Extracellular RoF was quantified using the photometer Ultrospec 7500 (Biochrom Ltd.) set at a wavelength of 509 nm (Mora‐Lugo et al. 2019).

## 3 Results

### 3.1 Overexpression of *ribM* Gene and Increased RF Production Alleviate the Antimicrobial Effect of RoF in *C. glutamicum*

**FIGURE 2:**
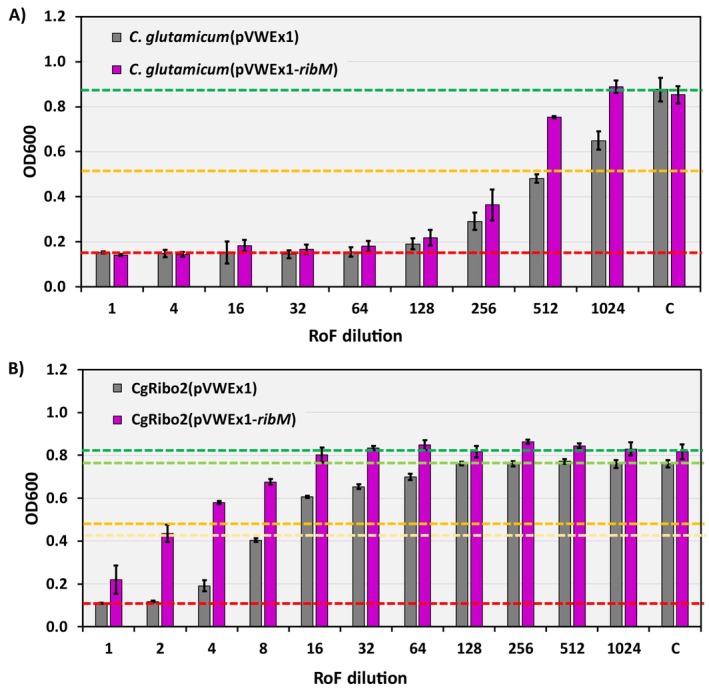
(A) Antimicrobial test with the strains *C. glutamicum* (pVWEx1) (grey bars) and *C. glutamicum* (pVWEx1‐*ribM*) (purple bars) to evaluate the effect of expressing the flavin transport gene *ribM* from *S. davaonensis*. (B) Antimicrobial test with the strains CgRibo2(pVWEx1) (grey bars) and CgRibo2(pVWEx1‐*ribM*) (purple bars) to evaluate the effect of RF overproduction. The RoF concentrations ranged between 500 mg/L (dilution 1) and 0.5 mg/L (dilution 1024). As a control (C), bacterial strains were grown with no RoF supplementation. Red dotted lines indicate complete inhibition of growth in plots A and B. Green dotted lines indicate the absence of growth inhibition in plot A and for the strain CgRibo2(pVWEx1‐*ribM*) in plot B. Yellow dotted lines indicate growth inhibition of 50% in plots A and for the strain CgRibo2(pVWEx1‐*ribM*) in plot B. Light green dotted lines indicate the absence of growth inhibition for the strain CgRibo2(pVWEx1) in plot B. Light yellow dotted lines indicate growth inhibition of 50% for the strain CgRibo2(pVWEx1) in plot B. Average and standard deviation values from biological triplicates are shown.

The membrane protein encoded by *ribM* enables RF uptake and RoF export, providing a protective mechanism against the toxicity of RoF in *S. davaonensis* (Hemberger et al. 2011). Here, the *ribM* gene was overexpressed in both the *C. glutamicum* wild‐type and the *C. glutamicum* RF overproducing strain CgRibo2 (Pérez‐García et al. 2022). As control strains, *C. glutamicum* wild‐type and CgRibo2 were transformed with the empty plasmid pVWEx1. The newly constructed *C. glutamicum* strains were evaluated in antimicrobial assays using minimal medium supplemented with decreasing concentrations of RoF from 500 mg/L (dilution 1) to 0.5 mg/L (dilution 1024) compared to the control condition (C) with no RoF supplementation (Figure 2). Overexpression of *ribM* in *C. glutamicum* wild‐type resulted in improved growth under 1 and 0.5 mg/L RoF (dilutions 512 and 1024, respectively) (Figure 2A). In contrast, RF overproduction led to an increased RoF tolerance, as the CgRibo2(pVWEx1) strain achieved biomass levels comparable to the control condition (0 mg/L RoF) across growth under 0.5 mg/L (dilution 1024) to 3.9 mg/L (dilution 128) of RoF (Figure 2B). Finally, overexpression of the *ribM* gene in CgRibo2 demonstrated an added positive effect, as the strain CgRibo2(pVWEx1‐*ribM*) achieved biomass levels comparable to the control condition when supplemented with approximately 31 mg/L (dilution 16) to 0.5 mg/L (dilution 1024) of RoF (Figure 2B). Remarkably, the strain CgRibo2(pVWEx1‐*ribM*) exhibited some level of growth across all tested RoF concentrations (Figure 2B). Hence, expression of *ribM* in an RF overproducing host has been proven to be beneficial for establishing RoF‐producing strains.

### 3.2 The Induction of *rosAB* Genes From *S. davaonensis* in *C. glutamicum* During Mid‐Exponential Growth Phase Enables Efficient RoF Production

**FIGURE 3:**
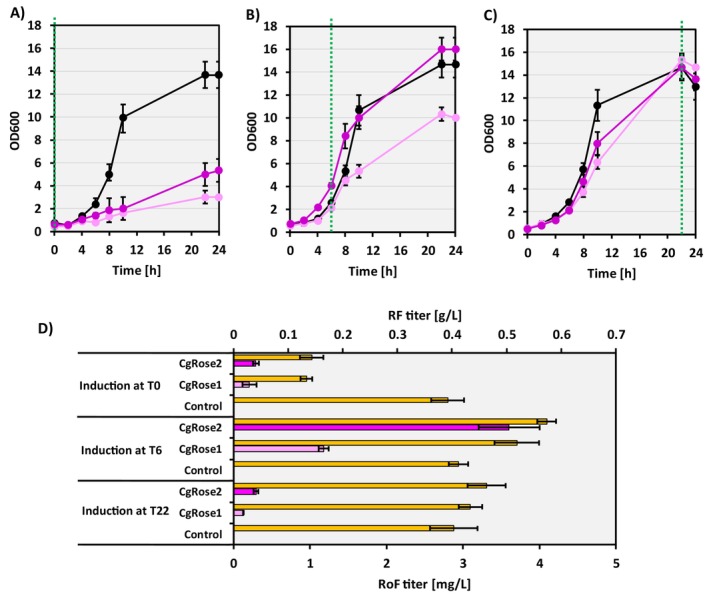
Growth curves of the strains CgRose1 (light pink lines), CgRose2 (dark pink lines) and the control strain (CgRibo2(pVWEx1); black lines) grown in 1% glucose minimal medium, with gene induction at the time points T0 (A), T6 (B) and T22 (C). The induction times are marked with dotted green lines. (D) RF (yellow bars) and RoF titers of the strains CgRose1 (light pink bars) and CgRose2 (dark pink bars) after growing the strains in 1% glucose minimal medium with different gene induction times. Average and standard deviation values from biological triplicates are shown.

Here, the RoF biosynthetic genes *rosA* and *rosB* from *S. davaonensis* were overexpressed both with and without co‐expression of *ribM* in the *C. glutamicum* RF overproducing strain CgRibo2 (Pérez‐García et al. 2022). The newly constructed strains CgRibo2(pVWEx1‐*rosAB*) and CgRibo2(pVWEx1‐*rosAB‐ribM*) were named CgRose1 and CgRose2, respectively. Next, a growth experiment was conducted in CGXII minimal medium using glucose as the sole carbon source, with the strain CgRibo2(pVWEx1) as a control strain. To evaluate the effect of induction on RoF production, IPTG inducer was added either at the start of the experiment (T0), during mid‐exponential phase after 6 h of incubation (T6), or in the stationary phase after 22 h of incubation (T22). Growth data were monitored for 24 h, and RF and RoF production data were collected after 24 h of cultivation (Figure 3). When heterologous gene expression was induced at T0, both CgRose1 and CgRose2 strains exhibited impaired growth compared to the control strain. However, by the end of the cultivation, CgRose2 accumulated significantly more biomass than CgRose1 (Figure 3A). When the inducer was added at T6, both CgRose1 and CgRose2 exhibited improved growth compared to induction at T0. Notably, the strain CgRose2, which expresses the *ribM* gene, showed growth comparable to the control strain (Figure 3B). This indicates that delaying induction mitigates the antimicrobial effect of RoF produced by the engineered strain. Interestingly, upon adding the inducer at T22, some growth impairment in both CgRose1 and CgRose2 was observed at approximately 10 h of growth. This may suggest leaky expression from the plasmid pVWEx1 (Figure 3C).

RoF accumulation in the supernatant was observed for the strains CgRose1 and CgRose2. Notably, the highest RoF titers were obtained when induction occurred during the mid‐exponential growth phase (T6). Specifically, the strain CgRose2 produced 3.6 ± 0.4 mg/L of RoF after 22 h of cultivation (Figure 3D). Surprisingly, RF production by the strains CgRose1 and CgRose2, when induced at T6, also appeared to improve by approximately 1.3–1.4‐fold (Figure 3D). To investigate the effect of *rosC* expression on RoF production, the strains CgRibo2(pVWEx1‐*rosABC*) and CgRibo2(pVWEx1‐*rosABC*‐*ribM*) were constructed and named CgRose3 and CgRose4, respectively. The strains CgRose1, CgRose2, CgRose3 and CgRose4 were subsequently cultivated in minimal medium with glucose as the sole carbon source, with IPTG added at T6. Surprisingly, expression of the *rosC* gene from *S. davaonensis* was found to negatively affect RoF production in *C. glutamicum* (Figure S1). CgRose3 and CgRose4 produced RoF titers of 1.6 ± 0.2 and 2.2 ± 0.3 mg/L, respectively, whereas the strains lacking *rosC*, CgRose1 and CgRose2, achieved higher titers of 2.0 ± 0.2 mg/L and 4.0 ± 0.1 mg/L, respectively (Figure S1). Hence, further research was conducted using the strain CgRose2.

### 3.3 The Expression of the Bifunctional RF Kinase Gene From *S. davaonensis* Enhances RoF Production in Engineered *C. glutamicum*

**FIGURE 4:**
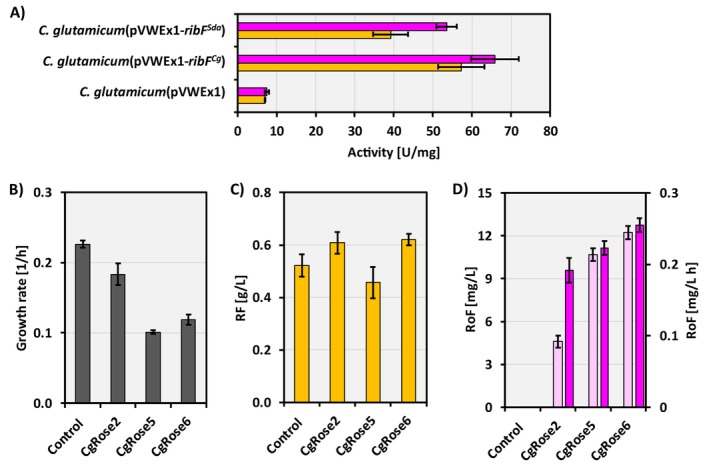
(A) Specific kinase activity in U/mg of the strains *C. glutamicum* (pVWEx1), *C. glutamicum* (pVWEx1‐*ribF* ^*Cg*^) and *C. glutamicum* (pVWEx1‐*ribF* ^*Sda*^) in the presence of RF (yellow bars) or RoF (pink bars). Growth rates (B), RF titers (C) and RoF production values (D) of the strains CgRose2, CgRose5, CgRose6 and Control (CgRibo2(pVWEx1)) grown in 1% glucose minimal medium. Gene expression was induced at T6. RoF production values are presented as titers (light pink bars) and volumetric productivities (dark pink bars). Average and standard deviation values from biological triplicates are shown. The statistical significance of the results is provided in Tables S2 and S3.

Further research focused on the gene *ribF* as a connector between the RF and RoF biosynthetic pathways (Figure 1). The *ribF* gene originating from *C. glutamicum* (*ribF* ^*Cg*^) or *S. davaonensis* (*ribF* ^*Sda*^) was cloned into the plasmid pVWEx1. The resulting plasmids pVWEx1*‐ribF* ^*Cg*^ and pVWEx1‐*ribF* ^*Sda*^, as well as the empty vector pVWEx1, were subsequently transferred into the *C. glutamicum* wild‐type strain. The resulting strains *C. glutamicum* (pVWEx1), *C. glutamicum* (pVWEx1‐*ribF* ^*Cg*^) and *C. glutamicum* (pVWEx1‐*ribF* ^*Sda*^) were grown in minimal medium and harvested during mid‐exponential phase. The biomass was lysed to create crude extracts containing the heterologous enzymes of interest. The kinase enzyme activities in *C. glutamicum* strains harbouring the *ribF* genes were determined in the presence of RF or RoF using the crude cell extracts (Figure 4A). While overexpression of both *ribF* genes resulted in kinase activity when RF or RoF was added to the reaction mix, the activity of RibF^Sda^ was significantly lower in the presence of RoF (53.5 ± 3.6 U/mg) compared to RibF^Cg^ (65.9 ± 6.0 U/mg). Similarly, RibF^Sda^ also showed lower kinase activity in the presence of RF as compared to RibF^Cg^ (39.2 ± 3.1 U/mg and 57.3 ± 5.9 U/mg, respectively) (Figure 4A). Next, the genes *ribF* ^*Cg*^ and *ribF* ^*Sda*^ were cloned together with the *rosAB‐ribM* genes, yielding the plasmids pVWEx1‐*rosAB‐ribM‐ribF* ^*Cg*^ and pVWEx1‐*rosAB‐ribM‐ribF* ^*Sda*^. The newly constructed plasmids were used to generate the strains CgRose5 and CgRose6 (Table 1). Next, the strains CgRose2, CgRose5, CgRose6 and CgRibo2(pVWEx1), the latter referred to as the control, were tested in growth experiments in CGXII minimal medium using glucose as the carbon source, and gene expression was induced at T6. The immediate effect observed from the overexpression of the *ribF* genes was a decrease in growth rate from 0.18 ± 0.02 1/h in strain CgRose2 to 0.10 ± 0.00 1/h and 0.12 ± 0.01 1/h in strains CgRose5 and CgRose6, respectively (Figure 4B). As a consequence, production data were collected for the control and CgRose2 strains after 24 h, and for the CgRose5 and CgRose6 strains after 48 h (Figure 4B–D). Regarding RF production, CgRose5 showed a similar titer of 0.46 ± 0.06 g/L compared to the control strain, which produced 0.52 ± 0.04 g/L RF, while CgRose2 and CgRose6 exhibited significantly higher RF titers of 0.61 ± 0.04 and 0.62 ± 0.02 g/L, respectively (Figure 4C). More importantly, RoF titers were greatly increased by overexpressing the *ribF* genes. The RoF production in the strain CgRose2 peaked at 4.6 ± 0.4 mg/L after 24 h, corresponding to a volumetric productivity of 0.19 ± 0.01 mg/L h. In contrast, the titers of CgRose5 and CgRose6 reached 10.7 ± 0.5 mg/L and 12.2 ± 0.5 mg/L after 48 h, respectively, reflecting volumetric productivities of 0.22 ± 0.01 mg/L h and 0.25 ± 0.01 mg/L h (Figure 4D). Because RoF production ceased at different time points for the strains tested, volumetric productivities were determined at the respective time points at which each strain reached the production plateau. In conclusion, the expression of an RF kinase gene appeared to be a key factor in RoF production, although it adversely affected the growth of the *C. glutamicum* strains.

### 3.4 Supplementation With the Vitamin Thiamine Further Enhances RoF Production in Engineered *C. glutamicum*

**FIGURE 5:**
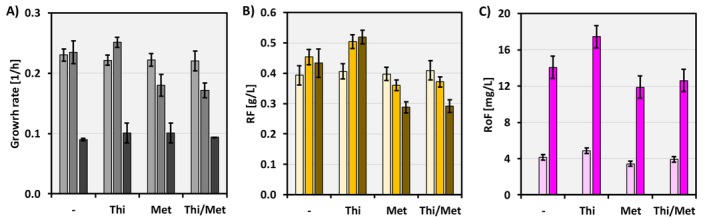
Growth rates (A), RF titers (B) and RoF titers (C) of the strains Control (CgRibo2(pVWEx1), light colours), CgRose2 (mid colour tones) and CgRose6 (dark colours). The strains were cultivated in minimal medium with 1% glucose as a carbon source, supplemented with 0.5 g/L thiamine HCl and/or 0.5 g/L methionine. RoF titers are only shown for the strains CgRose2 (light pink) and CgRose6 (dark pink). Average and standard deviation values from biological triplicates are shown. The statistical significance of the results is provided in Table S4.

The biochemical reactions from RF to RoF require the compounds thiamine and SAM (Schneider et al. 2020) (Figure 1). While thiamine is a vitamin that can be supplemented in the medium, the internal pool of SAM can be increased by supplementing the amino acid methionine (Kim et al. 2022). In this section of the study, thiamine HCl 0.5 g/L and/or methionine 0.5 g/L were supplemented in minimal medium with glucose as the carbon source, and the strains CgRibo2(pVWEx1) (referred to as the control), CgRose2 and CgRose6 were evaluated for growth and production (Figure 5). As previously, heterologous gene expression was induced at T6. RF and RoF titers were collected for the control and CgRose2 strains after 24 h and for the CgRose6 strain after 48 h. Under these conditions, and regarding strain growth, supplementation with thiamine did not result in any significant change compared to growth without thiamine. On the other hand, methionine supplementation had a negative impact on the growth rate of the control and CgRose2 strains. In particular, the growth rate of CgRose2 decreased from 0.23 ± 0.02 1/h in regular medium to 0.18 ± 0.02 1/h and 0.17 ± 0.01 1/h in minimal medium supplemented with methionine or with both thiamine and methionine respectively (Figure 5A). However, thiamine supplementation showed a positive effect on RF and RoF production in the CgRose2 and CgRose6 strains in comparison to the control. In the presence of thiamine, 11% more RF was produced by CgRose2 and 20% by CgRose6 (Figure 5B). Similarly, RoF production by the CgRose2 and CgRose6 strains increased by 17% and 24%, respectively, when thiamine was added to the minimal medium (Figure 5C). Accordingly, the final RoF production in this study peaked at 17.4 ± 1.5 mg/L, with a volumetric productivity of 0.36 ± 0.03 mg/L·h.

## 4 Discussion

Nowadays, new antimicrobial molecules and novel methods for producing them are being explored as potential alternatives to conventional antibiotics (Ajulo and Awosile 2024; Theuretzbacher et al. 2025). In particular, the use of microbial cell factories opens up promising opportunities in this field. Several studies have been conducted using bacteria like *B. subtilis* , *E. coli* and *C. glutamicum*. For instance, the production of mycosubtilin, an antifungal compound, was optimised in *B. subtilis* through promoter engineering (Leclère et al. 2005). In another study, also involving *B. subtilis* , the authors used CRISPR‐Cas9 to engineer the bacterium to produce fengycin, an antimicrobial lipopeptide, using xylose as the carbon source (Yin et al. 2024). Metabolic engineering of *E. coli* to produce flavonoids, specifically pinocembrin‐derived compounds known for their antimicrobial properties, has been achieved (Hanko et al. 2024). By introducing and optimising specific genes involved in the phenylpropanoid pathway, engineered *E. coli* strains achieved significant production levels of ferulic acid (Lv et al. 2021).

*C. glutamicum* demonstrates notable tolerance to a range of antimicrobial agents, enhancing its suitability for their production. For instance, its erythromycin resistance gene cassette has been shown to render *E. coli* resistant to erythromycin, tetracycline, puromycin and bleomycin (Jäger et al. 1997). Additionally, the MarR‐family transcriptional regulator CarR in *C. glutamicum* modulates resistance to antibiotics such as penicillin and streptomycin by activating the expression of genes involved in peptidoglycan synthesis, thereby strengthening the cell wall and enhancing stress resistance (Si et al. 2019). *C. glutamicum* naturally takes up arsenic compounds and possesses functional arsenite and arsenate reductases, tolerating concentrations up to 12 mM arsenite (Ordóñez et al. 2005). Furthermore, the bacterium robustly tolerates nalidixic acid, which has been historically used in transconjugation protocols to counter‐select *E. coli* donor cells based on its inhibitory activity against Gram‐negative bacteria (Hane 1971). *C. glutamicum* has also been engineered to produce antimicrobial molecules. Pediocin production was demonstrated in *C. glutamicum* via overexpression of the codon‐optimised pediocin biosynthetic operon *pedACD* ^*Cg*^, using genetic material from *Pediococcus acidilactici* PAC1.0 (Goldbeck et al. 2021). Similarly, the synthetic and codon‐optimised *nisZBTC* ^*Cg*^ operon was created based on genetic material from *Lactococcus lactis* and overexpressed in *C. glutamicum* to establish nisin production (Weixler et al. 2022). Recently, the *garQ* gene cluster from *Lactococcus garvieae* was codon‐optimised for *C. glutamicum* and its overexpression led to higher yields of the bacteriocin garvicin Q (Desiderato et al. 2024).

Here, we focused on RoF as a promising candidate as an antimicrobial. RoF inhibits the growth of Gram‐positive bacteria such as *E. faecalis* , *Streptococcus pyogenes* (Wang et al. 2017), *S. aureus* (MIC 1.25 mg/L) (Otani et al. 1980) and *L. monocytogenes* (MIC 0.5 mg/L) (Matern et al. 2016) as well as the protozoal parasites such as *Leishmania mexicana*, *Trypanosoma cruzi* and *Trypanosoma brucei* (Krajewski et al. 2017). Inside the bacterial cell, RoF is converted to RoFMN and RoFAD, which compromise the role of flavoproteins regarding cell function and growth (Langer et al. 2013). The natural RoF producer *S. davaonensis* possesses several defense mechanisms to mitigate the antimicrobial effect of RoF. The first one is the transport protein RibM, which participates in the uptake of RF and the secretion of RoF (Hemberger et al. 2011). Another mechanism is the FMN riboswitch in *S. davaonensis*, which is nonligand to RoFMN, making the riboflavin production unaffected by high RoF concentrations (Mora‐Lugo et al. 2019). Additional mechanisms include the slow reaction rate of the RosA enzyme, with a turnover rate of 0.06 1/min for the overall dimethylation reaction, and its 10‐fold tighter binding to its products, RoF and SAH, compared to AF and SAM (Tongsook et al. 2016). Similarly, the enzyme RosB also tightly binds to its reaction product AFP (Konjik et al. 2017). Our results showed that the expression of the *ribM* gene and/or overproduction of RF greatly mitigates the toxic effect of RoF. In particular, a high internal pool of RF appeared to be especially beneficial in this regard (Figure 1). This study also highlighted the importance of inducing RoF synthesis during the exponential phase (Figure 2). It appears that when the internal RF concentration reaches a certain level, it may ensure the production of sufficient FMN and FAD cofactors, thereby supporting the proper functioning of flavoproteins. Additionally, it may cover the lack of native RF biosynthesis caused by the RoF‐derived regulatory control of the FMN riboswitch in *C. glutamicum* . Overexpression of *rosAB* in *C. glutamicum* for the establishment of RoF was previously demonstrated (Mora‐Lugo et al. 2019). However, another gene, *rosC*, encoding a phosphatase, was later identified as the missing link in the RoF biosynthetic pathway (Joshi et al. 2024). Surprisingly, in this study, overexpression of *rosC* together with *rosAB* led to lower RoF production (Figure S1). RosC is a specific phosphatase that distinguishes between FMN and AFP, putatively avoiding potential metabolic drawbacks, as phosphorylation of RF by RibF using ATP, followed by inadvertent dephosphorylation of FMN by RosC, would create a futile cycle and lead to unnecessary energy expenditure in RoF‐producing strains (Joshi et al. 2024). This could indicate that the expression levels or gene order of the *rosABC* operon may influence RoF production in engineered *C. glutamicum* strains. Similarly, when establishing the production of the bacteriocin pediocin in *C. glutamicum* , the authors demonstrated the importance of the *ped* genes order (Goldbeck et al. 2021). In any case, *C. glutamicum* seems to possess a native phosphatase able to dephosphorylate AFP to AF. However, nucleotide and protein BLAST searches did not provide any clear genetic candidate; hence, more research needs to be done on this topic.

Regarding RibF from *S. davaonensis*, and according to previous studies, the enzyme is not RF‐specific and therefore is not involved in RoF resistance, as it can produce the modified flavin cofactors RoFMN and RoFAD (Grill et al. 2008). The flavokinase assay in this study indicated that both RibF kinases from *S. davaonensis* and *C. glutamicum* exhibited phosphorylation activity in the presence of RF and RoF (Figure 4A), suggesting substrate flexibility in this enzyme. However, the kinase activity of the *S. davaonensis* RibF enzyme was approximately 23% lower in the presence of RoF and 46% lower in the presence of RF compared to RibF from *C. glutamicum* . This reduction may have favoured the accumulation of RoF over its toxic phosphorylated analogs RoFMN and RoFAD in strain CgRose6. Despite higher RibF activity in CgRose5, the RF titer was lower than in CgRose6, which may be due to increased conversion of RF to FMN and FAD. Future investigations of nonsecreted metabolites will be required to fully understand this result. Also, RibF from *S. davaonensis* showed less kinase activity in the presence of RF compared with RoF. As previously reported, the catalytic efficiency of the RibF enzyme from *S. davaonensis* is significantly lower for RF (Km/Kcat = 0.0075 1/(μM·s)) as compared to RoF (Km/Kcat = 0.017 1/(μM·s)) (Grill et al. 2008). In this study, when overexpressing *ribF* genes in combination with RoF production, the growth rates were approximately halved (Figure 4A), which may indicate the presence of more toxic RoF‐derived cofactors, as the concentration of RoF produced also increased 2.7‐fold (Figure 4C).

Moreover, the supplementation of thiamine and methionine was also explored as it putatively boosts RoF biosynthesis reactions. Supplementing with methionine can enhance the formation of SAM (Kim et al. 2022; Luo et al. 2019), an important molecule involved in methylation processes essential for RosA‐mediated reactions. Yet, supplementation of methionine led to negative impacts on growth as well as RF and RoF production in the engineered RoF producers, but not in the control strain (CgRibo2(pVWEx1)) (Figure 5). In principle, supplementing energy‐intensive amino acids such as methionine can help conserve cellular energy (Graf et al. 2018), and previous studies have shown that methionine supplementation can be beneficial for *C. glutamicum* strains (Kim et al. 2022; Luo et al. 2019). On the other hand, exogenous methionine supplementation may lead to feedback inhibition in methionine synthesis pathways, altering the metabolic balance (Suda et al. 2008). Thus, the underlying reason for the effect observed in the RoF producers remains unclear. On the other hand, thiamine supplementation resulted in improved RoF production (Figure 5C). Thiamine supplementation in *C. glutamicum* is known to alleviate metabolic disturbances caused by iron deficiency, notably by reducing the secretion of pyruvate and 2‐oxoglutarate (Küberl et al. 2020). Thiamine supplementation in *C. glutamicum* research has been used to enhance the production of value‐added compounds like carotenoids and organic acids (Jeon et al. 2013; Taniguchi and Wendisch 2015). In this study, thiamine functions as an essential coenzyme for the RosB enzyme, which in turn directs the metabolic flux of FMN toward AFP (Schneider et al. 2020). Consequently, it is suggested that optimised RosB activity conditions resulted in increased RoF production, reaching the final titer value of 17.4 ± 1.54 mg/L. This RoF concentration represents a vast improvement compared to the previous RoF production using *C. glutamicum* strains, which was 1.6 ± 0.2 μM (0.56 ± 0.07 mg/L) (Mora‐Lugo et al. 2019). On the other hand, the recombinant *S. davaonensis RML7* produced up to 34.9 ± 5.2 μM (12.2 ± 1.8 mg/L) (Mora‐Lugo et al. 2019). The highest RoF titer reported to date is 129.6 mg/L, achieved with a recombinant *Komagataella phaffii* (formerly *Pichia pastoris*). This strain was developed by expressing the *rosA, rosB* and *rosC* genes from *S. davaonensis*, along with the FMN1 gene from *Candida famata* , under a constitutive promoter. The production was carried out in a 5‐L bioreactor over 13 days, with the maximum titer reached after 312 h of cultivation, meaning a volumetric productivity of 0.42 mg/L h (Dmytruk et al. 2023). In comparison, the CgRose6 strain engineered in this study achieved a RoF concentration of 17.4 ± 1.5 mg/L following 48 h of flask cultivation, corresponding to a volumetric productivity of 0.36 ± 0.03 mg/L·h. These results substantiate the capability of *C. glutamicum* strains to efficiently produce RoF.

## 5 Conclusions

This study demonstrates the potential of *C. glutamicum* as a host for RoF production. Overexpression of the RoF biosynthetic genes *rosAB*, the flavin transporter *ribM*, and the riboflavin kinase gene *ribF* in a riboflavin overproducing host resulted in the best‐performing strain in this study. The final strain CgRose6 achieved a RoF titer of 17.4 ± 1.5 mg/L and a competitive volumetric productivity value of 0.36 ± 0.03 mg/L·h in minimal glucose medium with thiamine supplementation.

## Author Contributions

**Luciana Fernandes Brito:** investigation, supervision, writing – original draft, writing – review and editing, methodology, conceptualization. **Ane Bræin Aas:** investigation, writing – review and editing. **Rosa Jodalen Rudberg:** investigation, writing – review and editing. **Trygve Brautaset:** writing – review and editing, supervision. **Fernando Pérez‐García:** investigation, writing – original draft, writing – review and editing, supervision, project administration, methodology, validation, conceptualization, funding acquisition.

## Conflicts of Interest

The authors declare no conflicts of interest.

## Supporting information


**Data S1:** Supporting Information.

## Data Availability

Data sharing is not applicable to this article as no datasets were generated or analyzed.
