# Supplementary material for: Production of the Antimicrobial Roseoflavin With Genetically Engineered Corynebacterium glutamicum
Source: Microb Biotechnol. 2025 Oct 22;18(10):e70246. doi: 10.1111/1751-7915.70246 (PMC12541554; doi:10.1111/1751-7915.70246)
Supplement: Supplementary file 1 — Data S1: Supporting Information. [file MBT2-18-e70246-s001.docx]

**Production of the antimicrobial roseoflavin with genetically engineered *Corynebacterium glutamicum***

**Luciana Fernandes Brito^1^, Ane Bræin Aas^1,^** ^+^**, Rosa Jodalen Rudberg^1,^** ^+^**, Trygve Brautaset^1^, Fernando** **Pérez-García^1, *^**

^1^Department of Biotechnology and Food Science, Faculty of Natural Sciences, NTNU, Trondheim, Norway

^+^Equal contribution

*** Correspondence:**Fernando Pérez-García
[fernando.perez-garcia@ntnu.no](mailto:fernando.perez-garcia@ntnu.no)

**Supplementary information:**

| **Table S1:** List of primers used in this work | | |
| --- | --- | --- |
| **Primer name** | **Sequence 5 > 3** | **Description** |
| ribMFw | **GCATGCCTGCAGGTCGACTCTAGAG**GAAAGGAGGCCCTTCAGGTGAACTGGCTCAACTCCGAGGC | Amplification of the *ribM* gene from *S. davaonensis* for the construction of plasmid pVWEx1-*ribM* |
| ribMRv | **AATTCGAGCTCGGTACCCGGGGATC**TCACGCGGGCGCTCCTTCCAGG |  |
| rosAFw | **GCATGCCTGCAGGTCGACTCTAGAG**GAAAGGAGGCCCTTCAGATGCGGCCGGAACCGACCGAGC | Amplification of the *rosA* gene from *S. davaonensis* for the construction of plasmid pVWEx1-*rosAB* |
| rosARv | TCAGCCGGCCGTGCCGCGGCATTC |  |
| rosBFw | **CGAATGCCGCGGCACGGCCGGCTGA***TTCGAACGCCCC*GAAAGGAGGCCCTTCAGATGGCTCTCAAGGCTCTCATTCTC | Amplification of the *rosB* gene from *S. davaonensis* for the construction of plasmid pVWEx1-*rosAB* |
| rosBRv | **AATTCGAGCTCGGTACCCGGGGATC**TCAGCCGAGTTGGCTCTCCTCGAC |  |
| rosBRv2 | TCAGCCGAGTTGGCTCTCCTCGAC | Amplification of the *rosB* gene from *S. davaonensis* together with the rosBFw primer for the construction of plasmid pVWEx1-*rosAB-ribM* |
| ribMFw2 | **GGTCGAGGAGAGCCAACTCGGCTGA***TTCGAACGCCCC*GAAAGGAGGCCCTTCAGGTGAACTGGCTCAACTCCGAGG | Amplification of the *ribM* gene from *S. davaonensis* together with the ribMRv primer for the construction of plasmid pVWEx1-*rosAB-ribM* |
| rosABFw | **AGCTTGCATGCCTGCAGGTCGACTCTAGAG**GAAAGGAGGCCCTTCAGATGCGGCCGGAACCGACCGAGC | Amplification of the *rosAB* genes from the plasmid pVWEx1-*rosAB* for the construction of plasmid pVWEx1-*rosABC* |
| rosABRv | **GAAAGCTCTCGCGTCCGTCACTCAC**CTGAAGGGCCTCCTTTC*GGGGCGTTCGAA*TCAGCCGAGTTGGCTCTCCTCGAC |  |
| rosCFw | **GGTCGAGGAGAGCCAACTCGGCTGA***TTCGAACGCCCC*GAAAGGAGGCCCTTCAGGTGAGTGACGGACGCGAGAGCTTTC | Amplification of the *rosC* gene from *S. davaonensis* for the construction of plasmid pVWEx1-*rosABC* |
| rosCRv | **CAGTGAATTCGAGCTCGGTACCCGGGGATC**TCAGATCACGTCGGACGGGGCCGCG |  |
| rosCRv2 | **AGGCCTCGGAGTTGAGCCAGTTCAC**CTGAAGGGCCTCCTTTC*GGGGCGTTCGAA*TCAGATCACGTCGGACGGGGCCGCG | Amplification of the *rosC* gene from *S. davaonensis* together with the rosCFw primer for the construction of plasmid pVWEx1-*rosABC-ribM* |
| ribMFw3 | **CGCGGCCCCGTCCGACGTGATCTGA***TTCGAACGCCCC*GAAAGGAGGCCCTTCAGGTGAACTGGCTCAACTCCGAGGCC | Amplification of the *ribM* gene from *S. davaonensis* together with the ribMRv primer for the construction of plasmid pVWEx1-*rosABC-ribM* |
| RibFCgFw | **GCATGCCTGCAGGTCGACTCTAGAG**GAAAGGAGGCCCTTCAGGTGGATATTTGGAGTGGACTAG | Amplification of the *ribF* gene from *C. glutamicum* for the construction of plasmid pVWEx1-*ribF^Cg^* |
| RibFCgRv | **AATTCGAGCTCGGTACCCGGGGATC**TTAAGCGCTGGGCTGGGTGTCG |  |
| RibFSdaFw | **GCATGCCTGCAGGTCGACTCTAGAG**GAAAGGAGGCCCTTCAGGTGCAGCGCTGGCGTGGCTTG | Amplification of the *ribF* gene from *S. davaonensis* for the construction of plasmid pVWEx1-*ribF^Sda^* |
| RibFSdaRv | **AATTCGAGCTCGGTACCCGGGGATC**TCAGCGGTCGCCCGCCTCC |  |
| ABMFw | **GCATGCCTGCAGGTCGACTCTAGAG**GAAAGGAGGCCCTTCAGATGCGGCCGGAACCGACCGAGC | Amplification of the *rosAB-ribM* genes from the plasmid pVWEx1-*rosAB-ribM* for the construction of plasmids pVWEx1-*rosAB-ribM-ribF^Cg^ and pVWEx1-rosAB-ribM-ribF^Sda^* |
| ABMRv | TCACGCGGGCGCTCCTTCCAGG |  |
| RibFCgFw2 | **CGTCCTGGAAGGAGCGCCCGCGTGA***TTCGAACGCCCC*GAAAGGAGGCCCTTCAGGTGGATATTTGGAGTGGACTAG | Amplification of the *ribF* gene from *C. glutamicum* for the construction of plasmid pVWEx1-*rosAB-ribM-ribF^Cg^* |
| RibFCgRv2 | **AATTCGAGCTCGGTACCCGGGGATC**TTAAGCGCTGGGCTGGGTGTCG |  |
| RibFSdaFw2 | **CGTCCTGGAAGGAGCGCCCGCGTGA***TTCGAACGCCCC*GAAAGGAGGCCCTTCAGGTGCAGCGCTGGCGTGGCTTG | Amplification of the *ribF* gene from *S. davaonensis* for the construction of plasmid pVWEx1-*rosAB-ribM-ribF^Sda^* |
| RibFSdaRv2 | **AATTCGAGCTCGGTACCCGGGGATC**TCAGCGGTCGCCCGCCTCC |  |
| Bold letters: Gibson assembly-directed overlapping sequences; underlined letters: ribosomal binding site sequences; italicized letters: linker sequences. | | |

**Table S2:** Specific kinase activity in U/mg of the strains *C. glutamicum*(pVWEx1), *C. glutamicum*(pVWEx1-*ribF*^Cg^), and *C. glutamicum*(pVWEx1-*ribF*^Sda^) in the presence of RF or RoF.

| **Strain** | **RF** | | | **RoF** | | |
| --- | --- | --- | --- | --- | --- | --- |
|  | Average | Std. Dev. | *p*-Value | Average | Std. Dev. | *p*-Value |
| *C. glutamicum*(pVWEx1) | 7.020 | 0.073 | - | 7.543 | 0.404 | - |
| *C. glutamicum*(pVWEx1-*ribF*^Cg^) | 57.300 | 5.903 | 0.007 | 65.900 | 6.001 | 0.005 |
| *C. glutamicum*(pVWEx1-*ribF*^Sda^) | 39.200 | 3.103 | 0.004 | 53.500 | 3.593 | 0.003 |

Values of average and standard deviation (Std. Dev.) from biological triplicates are shown. Averages were compared using Student’s t-test, and the corresponding *p*-values are shown for each engineered strain in comparison with the empty-vector control strain *C. glutamicum*(pVWEx1).

**Table S3:** Growth rates, RF titers, and RoF production and volumetric productivities values of the strains CgRose2, CgRose5, CgRose6, and Control (CgRibo2(pVWEx1)) cultivated in 1% glucose minimal medium.

| **Strain** | **Growth rate [1/h]** | | | **RF [g/L]** | | |
| --- | --- | --- | --- | --- | --- | --- |
|  | Average | Std. Dev. | *p*-Value | Average | Std. Dev. | *p*-Value |
| Control | 0.226 | 0.004 | - | 0.522 | 0.035 | - |
| CgRose2 | 0.183 | 0.012 | 0.018 | 0.609 | 0.033 | 0.000 |
| CgRose5 | 0.101 | 0.002 | 0.000 | 0.457 | 0.048 | 0.018 |
| CgRose6 | 0.119 | 0.006 | 0.000 | 0.621 | 0.018 | 0.015 |
| **Strain** | **RoF [mg/L]** | | | **RoF [mg/L h]** | | |
|  | Average | Std. Dev. | *p*-Value | Average | Std. Dev. | *p*-Value |
| Control | 0.000 | 0.000 | - | 0.000 | 0.000 | - |
| CgRose2 | 4.600 | 0.342 | 0.003 | 0.192 | 0.014 | 0.003 |
| CgRose5 | 10.695 | 0.371 | 0.001 | 0.223 | 0.008 | 0.001 |
| CgRose6 | 12.237 | 0.371 | 0.000 | 0.255 | 0.008 | 0.000 |

Values of average and standard deviation (Std. Dev.) from biological triplicates are shown. Averages were compared using Student’s t-test, and the corresponding *p*-values are shown for the strains CgRose2, CgRose5 and CgRose6 in comparison with the Control strain.

**Table S4:** Growth rates, RF titers, as well as RoF titers and volumetric productivities of *C. glutamicum* strains (Control strain CgRibo2(pVWEx1), CgRose2, and CgRose6) cultivated in minimal medium with 1% glucose as carbon source and supplemented with 0.5 g/L thiamine HCl and/or 0.5 g/L methionine.

| *Growth rate [1/h]* | | | | | | | | | |
| --- | --- | --- | --- | --- | --- | --- | --- | --- | --- |
|  | **Control** | | | **CgRose2** | | | **CgRose6** | | |
|  | Average | Std. Dev. | *p*-Value | Average | Std. Dev. | *p*-Value | Average | Std. Dev. | *p*-Value |
| - | 0.230 | 0.008 | - | 0.235 | 0.016 | 0.482 | 0.090 | 0.002 | 0.001 |
| Thi | 0.222 | 0.007 | - | 0.251 | 0.007 | 0.000 | 0.101 | 0.013 | 0.001 |
| Met | 0.222 | 0.009 | - | 0.180 | 0.015 | 0.010 | 0.101 | 0.013 | 0.001 |
| Thi/Met | 0.220 | 0.013 | - | 0.172 | 0.010 | 0.002 | 0.094 | 0.000 | 0.005 |
| *RF [g/L]* | | | | | | | | | |
|  | **Control** | | | **CgRose2** | | | **CgRose6** | | |
|  | Average | Std. Dev. | *p*-Value | Average | Std. Dev. | *p*-Value | Average | Std. Dev. | *p*-Value |
| - | 0.393 | 0.026 | - | 0.454 | 0.020 | 0.005 | 0.433 | 0.038 | 0.044 |
| Thi | 0.406 | 0.021 | - | 0.505 | 0.018 | 0.000 | 0.520 | 0.018 | 0.000 |
| Met | 0.398 | 0.018 | - | 0.360 | 0.014 | 0.005 | 0.288 | 0.015 | 0.000 |
| Thi/Met | 0.410 | 0.027 | - | 0.372 | 0.014 | 0.053 | 0.292 | 0.017 | 0.003 |
| *RoF [mg/L]* | | | | | | | | | |
|  | **Control** | | | **CgRose2** | | | **CgRose6** | | |
|  | Average | Std. Dev. | *p*-Value | Average | Std. Dev. | *p*-Value | Average | Std. Dev. | *p*-Value |
| - | 0.000 | 0.000 | - | 4.157 | 0.001 | 0.000 | 14.062 | 0.616 | 0.001 |
| Thi | 0.000 | 0.000 | - | 4.881 | 0.592 | 0.007 | 17.445 | 1.255 | 0.003 |
| Met | 0.000 | 0.000 | - | 3.432 | 0.001 | 0.000 | 11.888 | 0.904 | 0.003 |
| Thi/Met | 0.000 | 0.000 | - | 3.915 | 0.342 | 0.004 | 12.613 | 1.039 | 0.003 |

The values of average and standard deviation (Std. Dev.) from biological triplicates are shown. Averages were compared using Student’s t-test, and the corresponding *p*-values are shown for the strains CgRose2 and CgRose6 in comparison with the Control strain within each condition.

**Fig. S1:** Growth **(A)**, as well as RF **(B)** and RoF **(C)** production curves, of the strains CgRose1, CgRose2, CgRose3 and CgRose4 grown in 1% glucose CGXII minimal medium. Gene expression was induced at T6. Average and standard deviation values from biological triplicates are shown.
